# Biodegradable Hybrid Nanocomposite of Chitosan/Gelatin and Green Synthesized Zinc Oxide Nanoparticles for Food Packaging

**DOI:** 10.3390/foods9091143

**Published:** 2020-08-19

**Authors:** Santosh Kumar, Abhinab Mudai, Barnali Roy, Indra Bhusan Basumatary, Avik Mukherjee, Joydeep Dutta

**Affiliations:** 1 Department of Food Engineering and Technology, Central Institute of Technology Kokrajhar, Kokrajhar 783370, Assam, India; abhinabmudai1@gmail.com (A.M.); rbarnali700@gmail.com (B.R.); 023indra@gmail.com (I.B.B.); ak.mukherjee@cit.ac.in (A.M.); 2 Functional Materials, Department of Applied Physics, School of Engineering Sciences, KTH Royal Institute of Technology, AlbaNova Universitets Centrum, 106 91 Stockholm, Sweden

**Keywords:** green synthesis, ZnO NPs, biopolymer, active packaging, antimicrobial activity, composite films

## Abstract

In the context of emerging global concerns with synthetic plastic packaging, alternative natural biodegradable packaging materials are gaining increasing attention for food packaging applications. In this study, chitosan and gelatin nanocomposite hybrid films containing green synthesized zinc oxide (ZnO) nanoparticles (NPs) were developed and microstructural properties were studied. Antimicrobial activity of the developed films was evaluated using both Gram negative (*Escherichia coli*) and Gram positive bacteria (*Staphylococcus aureus*). Green synthesis protocol was used for the precipitation of ZnO NPs using fruit extract of *Cassia fistula*. The as-synthesized polyhedral ZnO NPs were in the range of 20–40 nm (average size ≈29 nm). Reinforcement with ZnO NPs in the hybrid films lead to improved thermal stability, elongation-at-break (EAB), and compactness properties. The developed films with 2% and 4% ZnO NPs showed a smooth, compact, and heterogeneous surface morphology compared to the control (chitosan-gelatin hybrid) films. Disc diffusion assays showed that the nanocomposite film had significant antimicrobial activity against *E*. *coli*. The developed hybrid nanocomposite films have potential to be developed as biodegradable alternative for postharvest packaging of fresh fruits and vegetables.

## 1. Introduction

Worldwide, fruits and vegetables are gaining consumers’ preferences, as these are healthy and rich in micronutrients. In India, as much as 40% of fresh produce are wasted during postharvest handling, storage, and transportation [1]. Physical injury during harvesting, microbial growth, and oxidative deterioration are primary causes of loss of freshness, quality, and shelf-life in fresh produce [2]. These necessitate strategies to enhance postharvest life in order to cater to their increasing global demands and longevity of the fresh products. Synthetic or plastic packaging of fresh fruits and vegetables is one of the most frequently used postharvest strategies applied to enhance the shelf-life. However, in the context of growing environmental concerns associated with synthetic plastic packaging, biodegradable alternatives are being sought for packaging of fresh produce. Natural biopolymers such as chitosan, agar, gelatin, zein, and starch are commonly used as alternatives to synthetic plastic packaging of food [3,4,5,6].

Chitosan is a linear polysaccharide of β-(1-4) linked D-glucosamine and N-acetyl glucosamine derived from chitin [2]. Chitosan has been found to be non-toxic, and approved by the United States Food and Drug Administration (US-FDA) as a generally recognized as safe (GRAS) material. Chitosan is abundantly available in exoskeletons of seafood such as crab, shrimps, locust, etc., and fish processing wastes [3]. Chitosan has been well-studied as a material for the fabrication of food packaging film due to its good film forming property, antimicrobial activity, biodegradability, low cost, and abundant availability [7]. Chitosan also possesses good mechanical properties, but suffers from high affinity towards moisture and lacks barrier property [8]. Mechanical and barrier properties of chitosan-based film can be improved by blending with other biopolymers and by reinforcing with nanomaterials. In recent years, chitosan-based nanocomposite films have been receiving a great deal of attention for their potential uses as biodegradable food packaging films [2,9]. Gelatin is a soluble animal protein derived mainly from collagen found in fibrous animal tissues. Gelatin composites with chitosan have shown improved physical, mechanical, and barrier properties of the film [9,10].

Metal and metal oxide nanoparticles commonly used in food packaging applications include silver (Ag), zinc oxide (ZnO), titanium dioxide (TiO_2_), and aluminum oxide (Al_2_O_3_) nanoparticles, as they provide food packaging with improved tensile strength, gas and UV barrier properties, ethylene-scavenging, and antimicrobial activities [11,12,13]. ZnO is one of the most suitable nano-materials for food application as they are GRAS material, and possess antimicrobial properties [14,15]. ZnO nanoparticles (ZnO NPs) were chemically obtained by precipitation by reducing zinc acetate in aqueous or organic media often stabilized using surfactants like cetrimonium bromide (CTAB) [16]. Typically ZnO NPs are synthesized by sol-gel or hydrothermal processes including green synthesis using plant extracts for the reduction of the zinc salt [17]. Green synthesis of ZnO nanoparticles (ZnO NPs) is preferred for food and biomedical applications, as physical and chemical synthesis involve toxic chemicals and extreme reaction conditions [18,19]. Plant extracts are mainly used for green synthesis of ZnO NPs, which act as a reducing as well as a stabilizing agent [19,20]. The green synthesis of ZnO NPs is inexpensive, easily scalable, and environmentally friendly [21].

In this work, we aimed to develop biodegradable films containing chitosan, gelatin, and green synthesized ZnO NPs at low costs. Variation in the thermal stability, mechanical integrity, and antimicrobial properties of the composite films obtained by the incorporation of ZnO NPs were studied to ascertain the suitability of the films in food packaging applications. Fruit extract of *Cassia fistula*, which is a medicinal plant possessing analgesic, anti-inflammatory, antidiabetic, as well as antioxidant activities was used for the green synthesis of ZnO NPs following a protocol described in the literature [22]. Extracts of *C. fistula* fruit consist of carbohydrates, phenolics, phospholipids, and amines that can serve as effective reducing agents for Zn^2+^ to facilitate the formation of ZnO NPs [23]. Mechanical properties of the developed nanocomposite films were characterized by texture analyzer, while thermal properties by thermogravimetric analyzer (TGA), surface morphology by scanning electron microscopy (SEM), and the chemical interaction among film constituents was studied with Fourier-transform infrared spectroscopy (FTIR) while the antimicrobial properties were characterized by disc-diffusion assays.

## 2. Materials and Methods

### 2.1. Materials

Fruit of *Cassia fistula* was collected from Tinali, a village in the vicinity of Central Institute of Technology Kokrajhar, BTAD, Assam (26°23′ N, 90°16′ E). Chitosan (≥90% deacetylation) and acetic acid were purchased from Research-Lab Fine Chem Industries, Mumbai, India and Avantor Performance Materials Ltd., Gurgaon, India, respectively. Purified gelatin and glycerol (98% purity) were procured from Merck Specialities Pvt. Ltd., Mumbai, India. Zinc nitrate hexahydrate (Zn(NO_3_)_2_*6H_2_O)with purity ≥96.0% was procured from Merck Life Science Pvt. Ltd., Bengaluru, Karnataka, India. The chemicals were used without any further purification.

### 2.2. Green Synthesis of ZnO Nanoparticles

The fruit of *Cassia fistula* was collected and washed with double distilled water (ddH_2_O). Pulp of the fruit was recovered by removing peel and seeds that were pulverized in a mixer grinder. In a typical extraction procedure, 25 g of pulverized pulp was suspended in 100 mL of ddH_2_O and brewing was carried out at 80 °C for 15 min. The solution was then filtered using Whatman filter paper no. 1 and the filtrate was stored in a glass container at ambient conditions until further use. For the green synthesis of ZnONPs, we adopted a previously used method with minor modifications [14]. In a typical synthesis protocol, 5 mL of aqueous fruit extract was taken in a glass container and diluted to a final volume of 25 mL using ddH_2_O. Then, 1 g of zinc nitrate hexahydrate (Zn(NO_3_)_2_*6H_2_O) was added, and the mixture was heated for 2 h at 60 °C with continuous stirring at 500 rpm. The resulting paste was washed several times with ethanol and ddH_2_O. The paste was then transferred to a ceramic crucible, and calcined at 400 °C in an atmospheric muffle furnace for 2 h. The calcined white solid was ground into powder form using a mortar, and was stored in an airtight container for further analysis and use. Similarly, synthesis of ZnONPs was also performed by using 10, 15, 20, and 25 mL of aqueous fruit extract in the reaction mixture. The control sample without aqueous fruit extract (by taking only 25 mL of ddH_2_O) was also prepared.

### 2.3. Characterization of ZnO NPs

The initial confirmation of ZnO NPs synthesis was achieved by UV-Vis spectrophotometry (Lambda-35, Perkin Elmer, Waltham, Massachusetts, USA) scanning in the range 200–800 nm. Aqueous suspensions of ZnO nano-powder were prepared for spectral analysis by sonication for 10 min using a bath sonicator. During nanoparticle synthesis, the concentration of the fruit extract was varied in the reaction mixture and the absorption spectra of the suspensions were measured as a function of reaction time. The morphology and particle size of as-synthesized ZnO NPs were imaged using high-resolution transmission electron microscope (HR-TEM) (G2 20, FEI Tecnai, Hillsboro, Oregon, USA) at an acceleration voltage of 200 keV. TEM analysis was performed only for the sample prepared with 10 mL of fruit extract wherein a drop of aqueous suspension of the prepared colloid was placed onto a copper grid of 300 meshes and allowed to dry in atmospheric conditions for 10 min for TEM microscopy studies.

### 2.4. Fabrication of Hybrid Nanocomposite Film

The film formation was achieved by following a method previously described by Kumar et al., with minor adaptations [9]. In total, 6 g of chitosan was dissolved in 300 mL of 1% (*v/v*) acetic acid using a magnetic stirrer (REMI, Mumbai, India) at 500 rpm for 6 h to obtain a (2%, *w/v*) chitosan solution. Similarly, 100 mL gelatin solution (4%, *w/v*) was homogenized by continuous stirring. Blending of the solutions (3:1, *v/v*) was done by stirring for 30 min. Then, 100 mL of the blended solution was incorporated with the ZnO NPs at 1%, 2%, and 4% separately, with respect to the amount of solid matter. Moreover, 30% (*w/w*) glycerol was added into each solution, and was stirred at 500 rpm for 15 min. Then, 15 mL of the nanocomposite film forming solution was casted in a glass Petri-dish and was allowed to dry for 2 days at ambient conditions. A control film without ZnO NPs was also prepared in a similar manner. The films were removed from the Petri-dish and stored in zip-lock plastic bags at 30 °C in 70% relative humidity until further use.

### 2.5. Characterization of Nanocomposite Films

#### 2.5.1. Thickness and Mechanical Properties

A digital micrometer (MitutoyoCorporation, Japan) was used to measure the thickness of the developed hybrid films. Five replicates as well as five different positions were considered for each film sample during thickness measurements. Mechanical properties i.e., tensile strength (TS) and percentage elongation-at-break (EAB) of the nanocomposite films were determined by using texture analyzer (TA.XTplus, Stable Micro Systems Ltd., Godalming, UK). The film samples were cut into pieces of 2.54 × 6.0 cm^2^ and the standard ASTM (American Society for Testing and Materials) method for the analysis was followed wherein 1 mm/min crosshead speed with 25 mm initial grip separation was used and for each film sample, five measurements were done and the average value is reported.

#### 2.5.2. Microstructural Analysis

Scanning electron microscopy (SEM) (Supra55, Carl Zeiss, Oberkochen, Germany) was used to study the surface morphology and microstructure of the hybrid films. A small piece of film sample was fixed on a SEM specimen holder using carbon tape and coated with sputtered gold film prior to its examination in the SEM. The surface morphology of the hybrid films, presence of nanoparticles and their size on the film surface were determined from the scanning electron micrographs.

#### 2.5.3. Infrared Spectroscopy

The presence of functional groups and molecular interactions among the various components of the hybrid films were determined using Fourier transform infrared spectroscopy (FTIR) spectroscopy (Spectrum-2, Perkin Elmer, Waltham, Massachusetts, USA) in the wavelength range of 4000–500 cm^−1^.

#### 2.5.4. Thermogravimetric Analysis (TGA)

The thermal stability of the hybrid nanocomposite films was determined by thermogravimetric analyzer (STA 6000, Perkin Elmer, Waltham, Massachusetts, USA). Approximately, 5 mg of the film sample was heated in a temperature range of 30–600 °C under nitrogen atmosphere (50 cm^3^/min) at a rate of 10 °C min^−1^, and weight loss of the sample was measured as a function of temperature.

### 2.6. Antimicrobial Analysis

Antimicrobial properties of the developed hybrid films were performed against *Escherichia coli* (*E. coli*) and *Staphylococcus aureus* (*S. aureus*) by agar disc-diffusion assay technique [24]. Inocula of the microorganisms were prepared from 24 h broth culture, and upon appropriate dilution to prepare 10^5^ CFU/mL sample, were spread over Mueller Hinton agar (MHA) surface. The UV radiated (for 20 min) disc (10 mm diameter) of the developed film was placed on the inoculated MHA surface. The diameters of the zone(s) of inhibition were determined after incubation of the plate for 24 h at 37 °C.

## 3. Results and Discussion

### 3.1. Characterization of ZnO Nanoparticles

**Figure 1:**
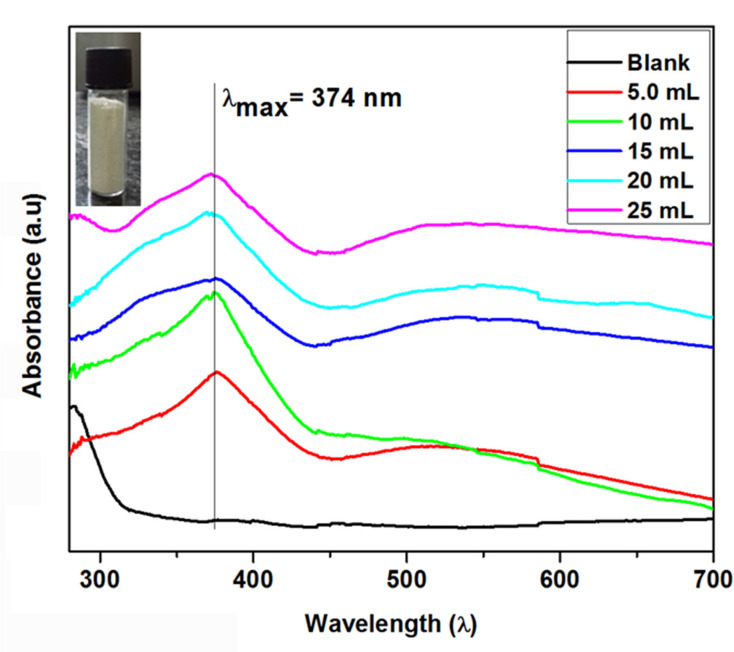
Optical spectra (UV-Vis) of colloidal zinc oxide (ZnO) nanoparticles (NPs) synthesized using *Cassia fistula* fruit extract (inset—camera photo of the synthesized ZnO NPs).

**Figure 2:**
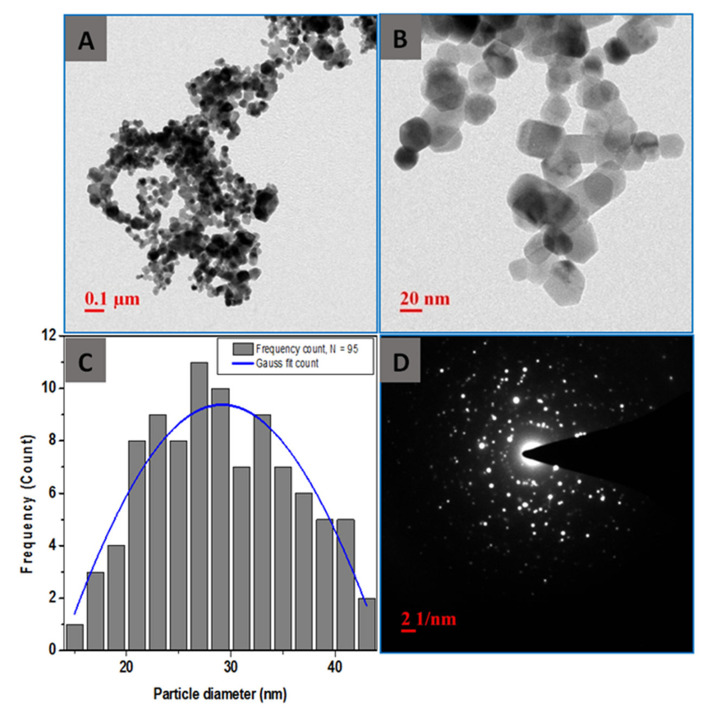
Transmission electron microscope (TEM) analysis of the as-synthesized ZnO NPs (**A**) showing the distribution of nanoparticles, (**B**) at a higher magnification showing the variation of particle sizes, (**C**) particle size distribution determined from 95 particles, and (**D**) selected area (electron) diffraction (SAED) pattern showing the polycrystallinity.

UV-Visible spectra of the synthesized ZnO nanoparticles showed maximum absorbance wavelength (λ_max_) at 374 ± 3 nm (Figure 1) confirming the presence of dispersed ZnO NPs in aqueous suspension [19,25]. The *C. fistula* fruit extract contains several phytochemicals such as polysaccharides, polyphenols, amino acids, alkaloids, terpenoids, etc., that can act as a reducing and stabilizing agent for the nanoparticles synthesis [26,27]. The intensity of the absorption peak of the samples usually decreased with the increase in amount of fruit extract in the reaction mixture. The sample with 10 mL of fruit extract showed sharp peak that corroborates to monodispersed ZnO NPs in the colloid [28]. The TEM images show that most of the nanoparticles are polyhedral in shape (Figure 2A,B). The diameters of the particles were found to be in the range of 20–40 nm with average diameter of about 29 nm (Figure 2C). The crystallinity was determined by selected area electron diffraction (SAED) pattern confirming the presence of polycrystalline ZnO NPs (Figure 2D).

### 3.2. Characterization of Hybrid Films

#### 3.2.1. Textural Properties

The thickness, tensile strength (TS), and percentage elongation-at-break (EAB) of the developed hybrid nanocomposite films are given in Table 1. The control film thickness was ≈84.53 µm, and incorporation of ZnO NPs increased thickness of nanocomposite films that is known to occur due to the increased viscosity of the nanocomposite solution [29]. The TS of the nanocomposite films decreased and EAB increased upon incorporation of ZnO NPs in the chitosan-gelatin matrix. The TS of the control film was 32.02 MPa, while for the films with 4% ZnO NPs it reduced to 26.39 MPa. Among the nanocomposite films, the films prepared with 2% ZnO NPs showed highest TS of 30.87 MPa. The significant decrease in TS of the films containing 4% ZnO NPs could be due to the formation of weak hydrogen bonds among gelatin, chitosan, and ZnO NPs. Upon ZnO NPs incorporation in the biopolymers, EAB of the films increased due to weakening of intermolecular hydrogen bonds between gelatin and chitosan, and formation of new hydrogen bonds between gelatin and ZnO NPs [30]. We have reported a similar reduction in TS and improvement in EAB in bionanocomposite films reinforced ZnO NPs [14], similar to what has been obtained by other researchers [31,32]. Higher EAB values are preferable for packaging films as it ensures better sealing, and improved load capacity of the packaging materials. Although, the incorporation of ZnO NPs in the chitosan-gelatin matrix decreased TS of the nanocomposite films down to 26.39 MPa, it is still comparable to synthetic polyethylene films (22–23 MPa) [33].

#### 3.2.2. Surface Morphology

**Figure 3:**
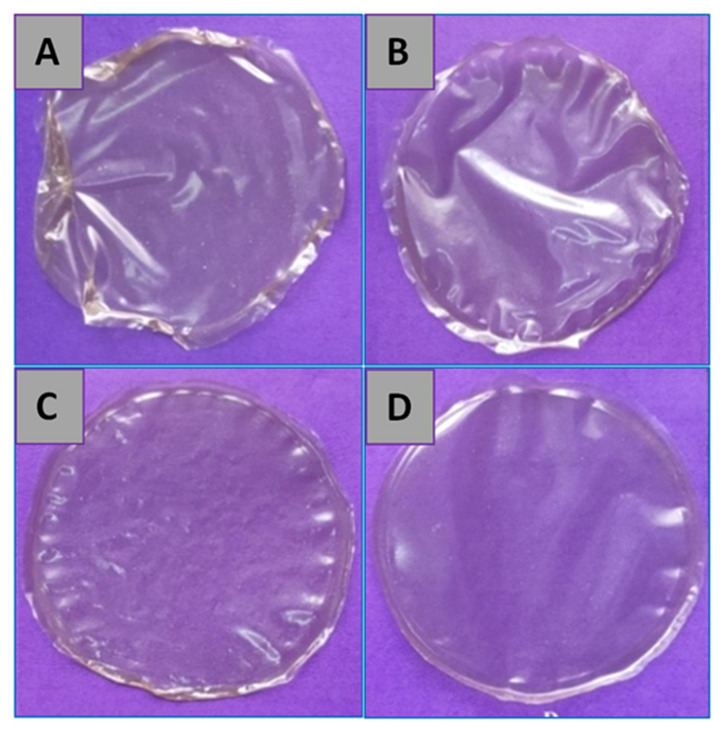
Photographs of the hybrid films; (**A**) chitosan (CH)/gelatin (GL) only, (**B**) CH/GL/ZnO (1%), (**C**) CH/GL/ZnO (2%), and (**D**) CH/GL/ZnO (4%).

**Figure 4:**
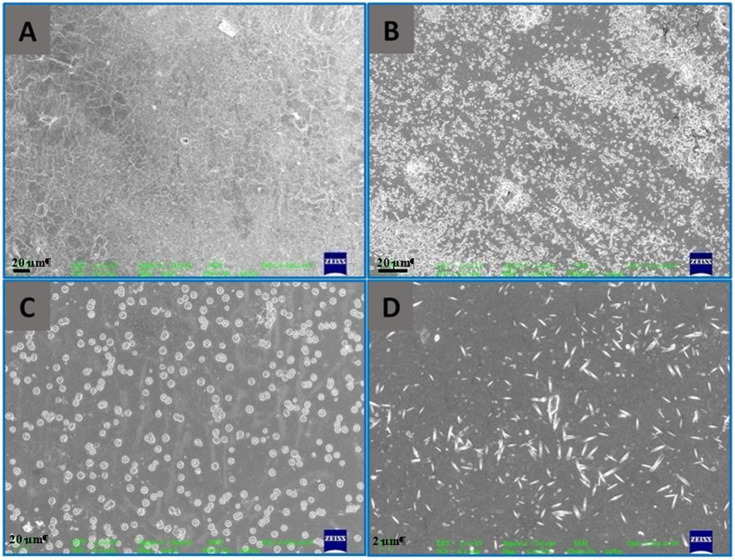
Scanning electron microscopy (SEM) micrographs of the developed hybrid films; (**A**) chitosan and gelatin film (CH/GL), (**B**) chitosan and gelatin with 1% ZnO NPs film (CH/GL/ZnO (1%)), (**C**) chitosan and gelatin with 2% ZnO NPs film (CH/GL/ZnO (2%)), and (**D**) chitosan and gelatin with 4% ZnO NPs film (CH/GL/ZnO (4%)).

The morphology of the hybrid nanocomposite film surfaces was studied by macroscopic and microscopic image analysis and the photographs of the developed films are presented in Figure 3. The films are translucent, and their appearance was not affected by the incorporation of ZnO NPs in the prepared nanocomposite films. The surface morphology and roughness of the hybrid films are shown in Figure 4. SEM micrographs showed that the hybrid films are compact, smooth, and heterogeneous compared to the control chitosan-gelatin films. Some cracks can be seen on the surface of control films (Figure 4A). The presence of the ZnO NPs on the surface of nanocomposite films are clearly visible and are evenly distributed (Figure 4B–D); similar to what was reported in previous studies [14,34]. The films prepared with higher ZnO concentrations (2% and 4%) showed some agglomeration of nanoparticles in the composite films. The agglomerates in the film containing 2% ZnO NPs were quasi-spherical (diameter ranges of ≈500–1000 nm) (Figure 4C), whereas they were rod shaped (diameter ranges of ≈200–400 nm) in the film containing 4% ZnO NPs (Figure 4D). Agglomeration and aggregation of nanoparticles due to van der Waals interaction is a well-known fact in colloid chemistry dependent on the concentration of ZnO NPs incorporated into the composite [32,35]. SEM results showed that the nanoparticles strongly adhere to the chitosan-gelatin matrix, which might be the reason behind improved physical and textural properties of the reinforced films.

#### 3.2.3. FTIR Analysis

**Figure 5:**
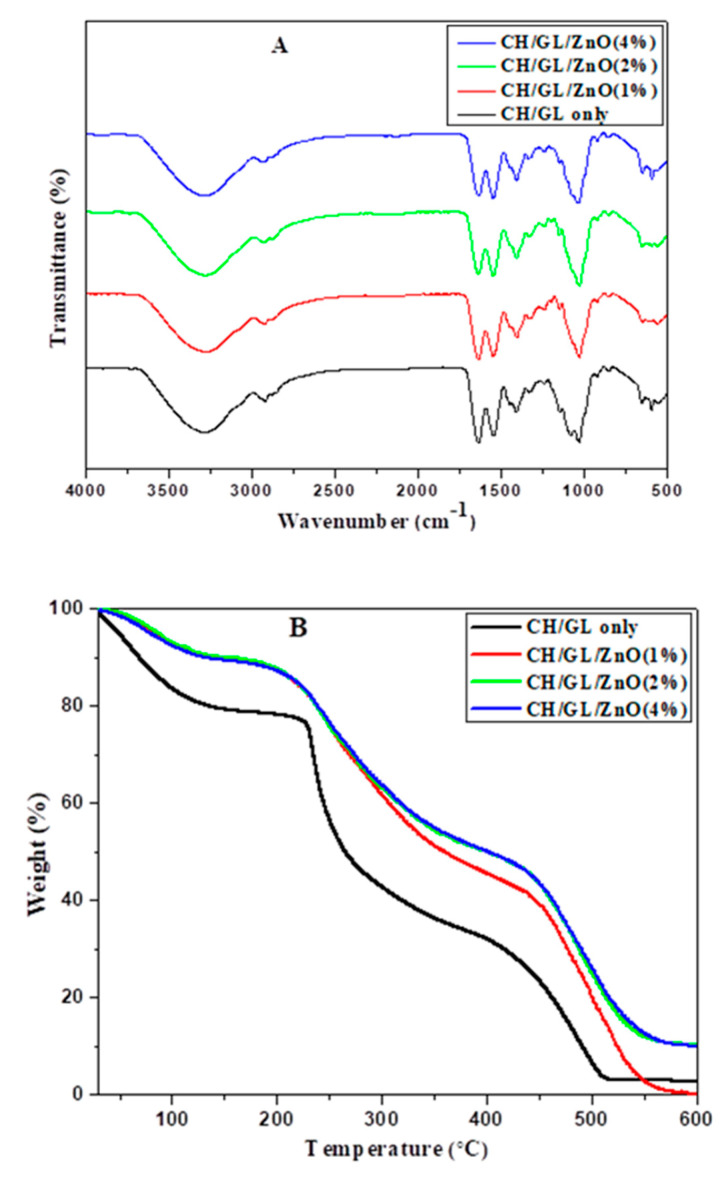
(**A**) Fourier-transform infrared spectroscopy (FTIR) spectra, and (**B**) thermogravimetric analyzer (TGA) thermograms of the developed hybrid films.

The FTIR spectra of the hybrid nanocomposite films reveals an interaction between the carbonyl group (-C=O) of chitosan and C-H group of gelatin (Figure 5A). The FTIR spectra of the control (CH/GL) sample film show strong absorption at 3293 cm^−1^ (-OH stretching), 2925 cm^−1^ (N-H stretching of amide A), 1549 cm^−1^ (C-C stretching), 1410cm^−1^ (C-O stretching), and 1640 cm^−1^ (C=O stretching) [36]. In addition, the absorption peak at ≈1035 cm^−1^ was can be attributed to the interaction between the -OH group of glycerol (used as a plasticizer) and gelatin in all the film samples [37]. The infra-red absorption peak positions and signal intensities changed upon incorporation of ZnO NPs in the nanocomposite films. Minor shift of the bands in the spectrum corresponding to hydroxyl, amino, and amide groups in the nanocomposite samples indicate the chemical interaction among the chitosan, gelatin, and ZnO nanoparticles [38]. Electrostatic interactions between the amino groups of chitosan (positively charged) and the negatively charged amino acid residues of gelatin are clearly observed from the FTIR spectra leading to the uniform distribution of ZnO NPs throughout the film, as observed in the SEM images.

#### 3.2.4. Thermal Stability

Results of thermal stability studies of hybrid nanocomposite films are shown in Figure 5B. The films show mainly four stages in the thermograms that might be related to moisture evaporation and decomposition of different components of the hybrid films. The initial weight loss of the films at ≈80–85 °C is due to moisture losses [39]. At this stage, the percentage of weight loss of the control (chitosan/gelatin) film was more (≈15%) compared to that of the nanocomposite films (≈7%), mainly because of different amount of moisture in the films since incorporation of ZnO NPs into chitosan-gelatin matrix increases compactness leading to a lower moisture content in the nanocomposite films [40]. A second weight loss was observed at ≈120–140 °C for control and at about ≈160–180 °C for the nanocomposite films. Weight loss at this stage may be due to evaporation of water adsorbed in the micropores and decomposition of glycerol [41]. The final weight loss was found at ≈360–370 °C in case of control film, and at 435, 460, and 460 °C in case of the nanocomposite films containing 1%, 2%, and 4% ZnO NPs, respectively, attributed to the decomposition of chitosan-gelatin matrices [38]. Noticeably, the incorporation of ZnO NPs increases thermal stability of the chitosan-gelatin hybrid films mainly due to increasing interaction between polymer chains hindering degradation of the polymeric matrices [42].

### 3.3. Antimicrobial Study

**Figure 6:**
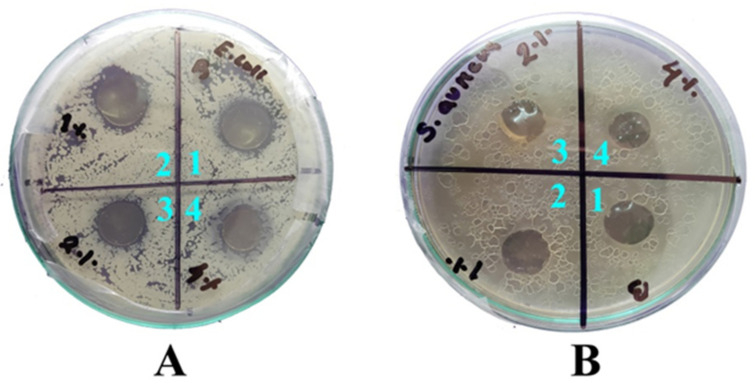
Antimicrobial activity of the films (1) CH/GL only, (2) CH/GL/ZnO (1%), (3) CH/GL/ZnO (2%), and (4) CH/GL/ZnO (4%) against (**A**) *Escherichia coli*, and (**B**) *Staphylococcus aureus*, after 24 h incubation.

The results of the antimicrobial study using the agar diffusion method for the developed hybrid nanocomposite films with *E. coli* and *S. aureus* as model organisms are given in Figure 6. The antimicrobial agent (ZnO NPs) diffuses into the agar inhibiting germination and growth of the test microorganism and the diameters of zones of inhibition are measured. The zones of inhibition of the developed hybrid films containing 1%, 2%, and 4% ZnO NPs were 10.5, 10.5, and 10.7 mm in diameter against *E. coli*, respectively (Figure 5A). A similar result was reported for chitosan based nanocomposite against *E. coli* [43]. The zone of inhibitions for *S. aureus* was not as prominent as that for *E. coli*. Chitosan has inherent antibacterial properties against several genera due to presence of positively charged amine groups that bind with negatively charged microbial cell membranes, leading to damage to the membrane hampering nutrient intake and causing leakage of cytoplasmic fluid [2,15,44]. ZnO NPs were incorporated into chitosan and gelatin matrix to further enhance their antimicrobial activity, due to the synergistic effect of the chitosan and ZnO NPs [45]. Haldorai and Shim (2013) reported that chitosan-ZnO nanocomposite inactivated 99.92% of viable *E. coli* within 24 h of in-vitro application [46]. In this study, it was also found that increased concentration of ZnO NPs led to increased antibacterial effect. The antimicrobial properties of ZnO NPs are mainly due to release of Zn^2+^ ions that strongly bind with the negatively charged bacterial cell wall and cause membrane damage [17]. Another mechanism is reactive oxygen species (ROS) generation resulting in oxidative stress to the cell and interaction with proteins, DNA, enzymes, and lipids inhibiting cell growth and/or cell death [47]. However, the antimicrobial effect may vary between Gram positive and Gram negative cells due to the difference in their cell wall structures [48,49].

The previous research work on development and application of nanocomposite films of chitosan/gelation/AgNPs and agar/ZnO NPs carried out by our group showed improved shelf-life of grapes for 10–15 days in ambient condition. This might be due to active migration of ZnO NPs from the film on to the packaged food [29]. In nanocomposite based active packaging, nanomaterials can migrate from the package, interact with the packaged food, and prevent or eliminate microbes from food. Thus, the developed hybrid film can also be potentially used as an antimicrobial active food packaging [9,14].

## 4. Conclusions

ZnO nanoparticles were synthesized through green chemistry using *Cassia fistula* fruit extract. The nanoparticles had polyhedral shape with average diameter of ≈29 nm. The synthesized ZnO NPs were reinforced into chitosan-gelatin biopolymer matrix, and were developed into nanocomposite hybrid films. The developed hybrid film showed improved elasticity, higher thermal stability, and enhanced structural integrity compared to the control films. In addition, the developed nanocomposite films had good antimicrobial activity against *E. coli*. Thus, chitosan-gelatin-ZnO nanocomposite films may be an effective antimicrobial defense and can be a promising alternative active packaging strategy to prolong shelf-life of packaged food.

## Figures and Tables

**Table 1:** Thickness and mechanical properties of the hybrid nanocomposite films.

Hybrid Films	Thickness (µm)	TS (MPa)	EAB (%)
CH/GL (control)	84.53 ^a^ ± 1.83	32.02 ^c^ ± 0.78	20.24 ^a^ ± 0.08
CH/GL/ZnO (1%)	86.35 ^a,b^ ± 2.97	29.45 ^b^ ± 0.59	24.03 ^b^ ± 0.29
CH/GL/ZnO (2%)	92.32 ^c^ ± 1.02	30.87 ^b^ ± 0.09	27.53 ^c^ ± 0.96
CH/GL/ZnO (4%)	88.61 ^b^ ± 0.98	26.39 ^a^ ± 0.72	35.65 ^d^ ± 0.71

values indicate the means ± standard deviation (*n* = 5); ^a, b, c, d^ indicate significant difference (*p* < 0.05).
